# Editorial: Multisystem inflammatory syndrome observed post-COVID-19: the role of natural products, medicinal plants and nutrients and the use of prediction tools supporting traditional forms of diagnosis

**DOI:** 10.3389/fphar.2025.1539793

**Published:** 2025-01-27

**Authors:** Anthony Booker, Ioannis Zabetakis, Nicole Elisabeth Teusch, Andrew Dalby, Thomas Hartung, Angela E. Peter

**Affiliations:** ^1^ Department of Ethnopharmacology & Medicinal Plant Science, University of Westminster, London, United Kingdom; ^2^ Department of Pharma & Biochemistry, UCL School of Pharmacy, London, United Kingdom; ^3^ Department of Biological Sciences, University of Limerick, Limerick, Ireland; ^4^ Department of Pharmaceutical Biology & Biotechnology, Heinrich-Heine-University Düsseldorf, Düsseldorf, Germany; ^5^ Department of Life Sciences, University of Westminster, London, United Kingdom; ^6^ Bloomberg School of Public Health, Johns Hopkins University, Baltimore, MD, United States; ^7^ Department of Biology, University of Konstanz, Konstanz, Germany; ^8^ Andhra University College of Science and Technology, Andhra University, Visakhapatnam, India

**Keywords:** multisystem inflammatory syndrome, COVID-19, natural products, medicinal plants, nutrition, prediction tools, health data science

The COVID-19 pandemic, caused by the SARS-CoV-2 virus, has had devastating global impacts since its emergence in late 2019. However, it also prompted an unprecedented scientific response (Cauchemez et al., 2024). While the acute phase of the pandemic has largely subsided due to quarantining, vaccination efforts and improved treatments, a new challenge has emerged in the form of post-COVID inflammatory syndromes. A hallmark of severe COVID-19 was excessive inflammation involving multiple organ systems, characterized by cytokine storms, coagulation dysfunction, and tissue damage. Even after recovery from acute infection, many patients continue to experience persistent inflammatory symptoms affecting various body systems, a condition now known as “long COVID” or “post-acute sequelae of SARS-CoV-2 infection” (PASC) (Barber et al., 2021; Davis et al., 2023; Liu et al., 2023; Porter et al., 2023).

The complex nature of post-COVID syndromes presents a significant challenge to healthcare systems worldwide. Symptoms can vary widely between patients and may affect multiple organ systems, including the respiratory, cardiovascular, neurological, and gastrointestinal systems. Common complaints include fatigue, cognitive dysfunction (“brain fog”), shortness of breath, anxiety, depression, and sleep disturbances. The underlying mechanisms of these persistent symptoms are not fully understood but are thought to involve ongoing inflammation, autonomic dysfunction, and potential autoimmune processes triggered by the initial SARS-CoV-2 infection.

Conventional medical approaches to treating post-COVID syndromes have shown limited success, highlighting the need for novel and integrative strategies. Natural products and plant-based medicines have a long history of use in treating inflammatory conditions and modulating immune function. Many of these compounds have well-documented anti-inflammatory, antioxidant, and immunomodulatory properties that could potentially address the multifaceted nature of post-COVID syndromes.

The global impact of long COVID cannot be overstated. With millions of people worldwide experiencing persistent symptoms following SARS-CoV-2 infection, the social, economic, and healthcare burdens are immense. The development of effective treatments for post-COVID syndromes is therefore of paramount importance. As we continue to navigate the long-term consequences of the COVID-19 pandemic, interdisciplinary collaboration between conventional and complementary medicine will be crucial. By combining the strengths of various therapeutic approaches and diagnostic methods, we may be better equipped to tackle the complex challenges posed by post-COVID syndromes. The studies in this Research Topic demonstrate the potential of such integrative approaches and pave the way for future research in this critical area.

This Research Topic explored innovative approaches to address post-COVID inflammatory syndromes using natural products, medicinal plants, nutrients, and integrative diagnostic methods. The articles in this collection investigate a range of natural compounds and plant-based medicines for their anti-inflammatory and immunomodulatory properties that may help alleviate lingering post-COVID symptoms. Additionally, the potential of nutritional interventions and traditional diagnostic techniques to support patients with post-COVID syndromes is examined.

The research presented in this collection explores several promising avenues for natural interventions. These include the use of specific vitamins and nutrients to support immune function and reduce inflammation, herbal extracts with known anti-inflammatory properties, and comprehensive lifestyle interventions addressing diet and physical activity. By targeting multiple aspects of post-COVID syndromes simultaneously, these integrative approaches may offer advantages over single-target pharmaceutical interventions.

Moreover, this Research Topic delves into the potential of traditional diagnostic methods to complement conventional techniques in assessing and monitoring patients with post-COVID syndromes. These approaches may provide valuable insights into the complex interplay of symptoms and underlying physiological imbalances, allowing for more personalized and holistic treatment strategies. As our understanding of post-COVID syndromes continues to evolve, this research provides valuable insights into natural therapeutic options that may work in concert with conventional treatments to improve outcomes for affected patients.

1. Deng et al. examined the relationship between fat-soluble vitamin status and antibody responses to COVID-19 vaccination in a cohort of 141 healthy adults. They found that higher plasma vitamin D levels were associated with lower anti-SARS-CoV-2 antibody titers, both for wild-type and Omicron variants. This unexpected finding suggests that vitamin D may play a complex role in modulating vaccine-induced immunity, highlighting the need for further research on optimal vitamin D levels for vaccine efficacy.
2. Pourfarzi et al. conducted a randomized controlled trial testing a web-based lifestyle intervention focused on nutrition and physical activity for preventing COVID-19. The study involved 303 women aged 30–60 who had not previously contracted COVID-19. The intervention group received online educational sessions on healthy diet and physical activity. After 4 weeks, the intervention group showed significant improvements in weight, BMI, nutritional status, and physical activity levels compared to controls. Importantly, the intervention group also had a lower incidence of COVID-19 infection during the follow-up period, suggesting that lifestyle modifications may help reduce COVID-19 risk.
3. Gaylis et al. evaluated a nutraceutical supplement containing multiple compounds, including β-caryophyllene, pregnenolone, and various herbs and vitamins, for treating long COVID symptoms. In an open-label trial with 51 participants, the supplement significantly improved various persistent symptoms including fatigue, weakness, cognitive issues, and shortness of breath over 4 weeks of treatment. The study demonstrated the potential of this multi-component natural approach in addressing the complex symptomatology of long COVID.
4. Joung et al. investigated the effects of a herbal extract (Myelophil) on fatigue symptoms in long COVID patients. The study was a non-randomized, open-label observational study, without a control group. Myelophil was administered for 4 weeks to the 49 participants (18 males, 31 females) in this study. After 4 weeks of Myelophil administration, participants showed significant improvements in fatigue scores, physical weakness, and quality of life measures. This study provides evidence for the potential efficacy of traditional herbal medicine in managing long COVID symptoms.
5. Bioinformatics and systems biology approaches were employed by Qian et al. for the identification of hub genes, shared pathways, molecular biomarkers, and candidate therapeutics for the management of sepsis and sepsis-induced ARDS in the context of COVID-19 infection. 189 differentially expressed genes (DEGs) shared among COVID-19 and sepsis datasets were identified. Construction of protein-protein interaction networks revealed that six hub genes (CD247, CD2, CD40LG, KLRB1, LCN2, RETN) exhibited significant alterations across COVID-19, sepsis, and geriatric sepsis-induced ARDS. Functional analysis underscored the interconnection between sepsis/sepsis-ARDS and COVID-19, enabling the identification of potential therapeutic targets, transcription factor-gene interactions, DEG-microRNA co-regulatory networks, and prospective drug and chemical compound interactions involving hub genes.
6. Qian and Zeng investigated the effects of Jinhua Qinggan Granules (JHQG), a Traditional Chinese Medicine formulation, on COVID-19 using mass spectrometry, network pharmacology, and single-cell RNA sequencing analysis. The researchers identified 73 chemical components in JHQG and constructed a network showing interactions between these compounds, target proteins, and immune cells. Results suggest JHQG may mitigate inflammation in COVID-19 by inhibiting the activity of activated neutrophils, monocytes, plasmoblasts, and effector T cells in peripheral blood. The findings provide insights into JHQG’s mechanism of action and support its potential as a safe and effective treatment for viral infections like COVID-19.
7. Ahmad et al. published the first survey reviewing the application of AI methodologies on Long COVID. Twenty research papers that met the inclusion criteria (innovative AI approach with clear results published after 2020) employing AI techniques such as ML and NLP on Long COVID data are discussed. Thirteen papers were focused on using ML techniques and the other seven were on applying text mining to Long COVID data. The data, AI techniques implemented, accuracy, and precision of each of the 20 papers are reviewed in detail. The use of AI techniques to analyze temporal data such as a symptom or to physiologically monitor data over time, can assist in detecting early signs of worsening of Long COVID, facilitating both timely medical interventions and personalized adaptation of treatment protocols hence improving patient outcome.

This Research Topic has made significant strides in exploring natural and integrative approaches to address the complex challenge of post-COVID inflammatory syndromes. The collected studies investigated a diverse range of interventions, from specific nutrients and herbal formulations to comprehensive lifestyle modifications. Collectively, these studies offer valuable insights into natural therapeutic options that may complement conventional treatments, potentially improving outcomes for those affected by post-COVID syndromes. The research underscores the importance of integrative approaches in addressing the multifaceted nature of long COVID and opens new avenues for future investigations in this critical area of public health.

It is important to note that while the results presented in these studies are encouraging, further research is needed to fully establish the efficacy and safety of these natural interventions for post-COVID syndromes. Large-scale, randomized controlled trials will be crucial in validating these findings and determining optimal treatment protocols. Additionally, the potential interactions between natural products and conventional medications must be carefully considered to ensure patient safety.
